# Prevalence of depression and associated factors among community hosted internally displaced people of Tigray; during war and siege

**DOI:** 10.1186/s12888-023-05333-3

**Published:** 2024-01-02

**Authors:** Aregawi Gebreyesus, Afewerki Tesfahunegn Niguse, Fiyori Shishay, Liya Mamo, Teklit Gebremedhin, Kahsu Tsegay, Asqual Gebreslasie Gebremariam, Kokob Gebru Kidanu, Solomon Gidey, Fisaha Tesfay

**Affiliations:** 1 Department of Epidemiology, College of Health Science, Mekelle University, Mekelle, Tigray Ethiopia; 2 Department of Biostatistics, College of Health Science, Mekelle University, Mekelle, Tigray Ethiopia; 3 School of Medicine, College of Health Science, Mekelle University, Mekelle, Tigray Ethiopia; 4 School of Nursing, College of Health Science, Mekelle University, Mekelle, Tigray Ethiopia; 5 Department of Psychiatry, College of Health Science, Mekelle University, Mekelle, Tigray Ethiopia; 6 Deakin University, Geelong, Institute for Health Transformation, Melbourne, Australia; 7 College of Medicine and Public Health, Flinders University, Bedford Park, Australia

**Keywords:** Depression, War, IDP, Tigray

## Abstract

**Background:**

Displacement is a psychologically stressful event. Since the war began, the people of Tigray were subjected to stressful events such as torture, rape, the killing of a family member, forced displacement, and even ethnic cleansing in their home countries. Especially displaced individuals are faced mental health problems. This study aimed to assess the prevalence of depression and associated factors among community internally displaced people due to the war on Tigray, in 2021.

**Methods:**

The community-based cross-sectional study design was applied from August 06 to 30, 2021 in all *Weredas* of Tigray. A total of 1,990 cIDP were recruited through a two-stage sampling technique. A structured and standardized questionnaire was used to collect data. Both bivariate and multivariable logistic regression was applied to identify associated risk factors and AOR with 95% confidence interval was used to select statistically significant variables.

**Results:**

The prevalence of depression among internally displaced people was 81.2% (95% CI: 79.4–83%), with more than 60% categorized as moderate and severe depression. The married and divorced marital status, being government employee, having family size >  = 4, destruction of household fixed assets, looting of cereals, and having disabled family members due to the war were the significantly associated risk factors of depression.

**Conclusions:**

The prevalence of depression among community internally displaced people during the war on Tigray is very high compared to other studies conducted elsewhere. Almost 8 of 10 IDPs are developed depression and this is a very series health issue that needs immediate intervention by local, international organizations and communities around the world.

## Background

War causes significant displacement of people due to the destruction of homes, environment, religious places, political persecution, and economic necessity. Internally displaced persons (IDPs) are persons who have been forced to flee their homes or places of usual residence as a result of armed conflicts, situations of generalized violence, violations of human rights, and disasters but remain within the borders of their own country. The number of internally displaced persons in the world has been increasing from time to time as a result of human-made or natural disasters [1, 2]. In 2022, more than 100 million people were displaced globally and 52.3% of them are internally displaced persons as a consequence of war and violence. The mass majority of the IDPs were from low-and middle-income countries [3].

Displacement is a psychologically stressful event [4]. The displaced individuals may be subject to stressful events such as torture, rape, assassination, and even ethnic cleansing in their home countries and throughout their journey [5]. Displaced survivors who experienced multiple war events perceived multiple negative effects of war on their life domains related to individuals who lived in war areas [6, 7]. Considering the effects, displacement may be accepted as a public health problem and a type of disaster, as it may lead to loss of resources, economic uncertainty, absence of health services and education, insouciant compensation for fundamental human requirements, and disintegration of public structure [5, 8]. IDPs, mainly those affected by wars, are in high danger of mental health problems. The frequently stated psychological responses are depression as a response to loss [2, 9, 10].

In different epidemiological surveys and studies on the psychopathology of war survivors depression is among the most prevalent mental health problems [5, 11]. Depression affects about 121 million people internationally. And it is one of the highest causes of disability globally [12]. Across different studies, the prevalence rates of depression and other mental health problems among war-affected populations vary widely. The prevalence of depression among people affected by armed conflicts ranges between 2.3 to 80% [5]. Studies conducted in Turkey and Afghanistan indicated that the prevalence of depression was 5% and 38.5% respectively [5]. The literature revealed that aggravating factors associated with depression among IDPs were factors such as gender, marital status, a distance of displacement, experiencing a lack of food or water, and experiencing sexual abuse [6, 13].

As a result of the war that began between the Tigray regional government and the Ethiopia federal government on Nov 04/2020, an estimated 2.1 million Tigreans have been internally displaced from their homes and over 61,000 Tigreans including children and women arrived in eastern Sudan days after the war [14–16]. Internally Displaced Persons (IDPs) are among the hardest hit by the war, most of who are hosted by the community while others are living in crowded collective centers [17]. As IDPs have left their homes forcibly they are less accessible by the international humanitarian agencies. They are in a more desperate situation than even refugees and remain at the forefront of the humanitarian agenda.

The war was waged by the Ethiopian government forces, Eritrean government forces, Amhara regional state special forces, and Amhara militia, against the regional government of Tigray [18, 19]. In Tigray's war, the non-combatant population had been agonized and tortured, were murdered and humiliated extensively in mass, were forcefully displaced with the abduction of their properties, battered, became forced laborers, sexually slaved (women and girls were raped in groups, raped in front of their families and relatives, the soldiers were inserting foreign bodies like nails, stones, sponges and other foreign substances into their genitalia) [17, 20–25].

The war in Tigray resulted in millions of displacements, starvation (due to the full blockade of humanitarian aid), and many thousand deaths. Due to that intolerable exposure to different stressors, mental health problems may last and affect the community at large. Even though there are high rates of IDPs due to the war in Tigray and the level of depression was not studied yet. Thus, this study aimed to assess the level of depression and associated factors among Community hosted Internally Displaced Persons (cIDPs) due to the war crisis in Tigray.

## Methods

### Study setting and design

This study was conducted using a community-based cross-sectional study design from August 06 to 30, 2021 in Tigray. Tigray was a regional state of Ethiopia that bordered with Amhara and Afar regions of Ethiopia in the south, Eritrea in the north and east, and Sudan in the west. Based on the USAID 2021 report Tigray had 2.1 million internally displaced persons due to the war launched by the government of Ethiopia [15].

### Study population

The study population of this study was all households (HH) heads of community hosted internally displaced persons hosted in a community of all zones of Tigray except the western zone. But HH heads who were not available after twice check-ups were excluded from the study and replaced with a nearby household of cIDPs.

### Sample size and sampling technique

The sample size was calculated based on the double proportion formula using the assumption of previously conducted studies with the variable of 'seriously injured'; with an odds ratio of 1.4, 60.8% percent of not seriously injured due to the war, and 1.5 design effect, and 5% non-response rate. Based on the assumption, the minimum sample size was 1,990 [13].

Two-stage sampling techniques were applied to select study participants. The sampling frame of households was received from the social affairs of the districts and towns. After selecting those households that hosted cIDPs, the displaced family's heads were interviewed for the assessment. When there were two or above family members in the same hosting HH, one family member was selected based on randomly.

### Data collection tool and technique

The interviewer-administered structured and standardized questionnaire was used to collect the data at the household level by trained data collectors and supervisors. The questionnaire had four parts: socio-demographic, economy-related questions, health-related questions, and a checklist for MDD based on the criteria of the Diagnostic Statistical Manual for Mental Health Disorders (DSM-IV). PHQ-9 checklist has nine items, each of which offers four response choices ranging from not at all (scores 0 points) to nearly every day (scores 3 points) with a total score of 27 [12, 26]. And the tool (PHQ-9) is validated in Ethiopia and showed good internal (Cronbach's alpha = 0.85) and test re-test reliability (intra-class correlation coefficient = 0.92) [27].

### Variables and measurements

The outcome variable of this study was having depression which was measured using PHQ-9 with a 3-point severity scale over the last two weeks. Based on the validated standard of PHQ-9 score ≥ 5 is considered to meet the symptom of depression. The instrument was incorporated with the Diagnostic and Statistical Manual for mental disorder version IV (DSM-IV) depression criteria with other leading depression symptoms into a brief self-report tool [12, 26, 27].

The independent variables were also, Socio-demographic characteristics: Age, marital status**, e**ducational status, occupation, and displaced family size**;** Economic-related variables: condition of fixed Assets, property loss during the war, shortage/Lack of necessities**,** looting of domestic animals, looting of Machineries/ House commodities and looting of cereals**;** Health-related variables: Losing contact with a family member**,** death of family member due to war, family member faced disability and family member experienced acute health problem.

Internally displaced persons are those who are displaced from their residential areas in Tigray due to the war in other areas within Tigray [28, 29].

### Data processing and analysis

After finishing data collection and coding, the data were entered using Epi-data 3.1 and then exported to SPSS V-26 for cleaning and analysis. First, descriptive analysis was done for the sociodemographic factors, economic factors, and health condition of the family using frequency with percentages and graphs for categorical variables. To determine the level of depression, respondents were categorized into no depression (scored less than 5 denoted “zero”) and having depression (scored more than or equal to 5 denoted “1”). The severity of depression was also ordered from minimum depression to severe depression denoted from “1” to “5” respectively.

Binary logistic regression was applied for identifying associated factors with depression. Variables that had < 0.25 P-value in bivariate analysis were entered into a multivariable logistic regression analysis to identify the associated independent variables. To minimize the effect of confounders, multi-Collinearity was checked by a variance inflation factor (VIF), and those with a VIF of < 10 was included. The fitness of the model was tested by Hosmer and Lemeshow's goodness-of-fit test and the value was 0.809. Finally, an Adjusted odds ratio (AOR) with a 95% confidence interval was used to show the strength of the association and a P-value < 0.05 was taken to declare the statistical significance of a variable.

### Ethical consideration

Ethical clearance was obtained from Mekelle University, College of Health Science Ethical Review Board on August 2, 2021, with IRB Ref: MU-IRB 1908/2021. The Ethics committee has also approved the informed verbal consent of participants. For those who can not read and write, the informed consent form was read to them by the interviewer and then requested to sign using finger stamp self-ink. Additional support letter from Tigray Health Bureau, *woreda* health offices, and verbal consent from the participant was assured before actual data collection. The HH heads and other eligible study participants were communicated and verbal consent was obtained before the data collection. To protect the participants, no individual identifying information was collected.

## Results

### Socio-demographic characteristics

A total of 1,965 participants were interviewed in this survey study with a response rate of 98.7%. The median age of the participants was 31 (IQR = 14) and more than half (55.3%) were female participants. Regarding the marital status and religion of the family head of displaced people who were hosted within the community of Tigray; 69% of them were married and 98% were orthodox Christianity followers (Table 1).

**Table 1:** Socio-demographic characteristics of cIDP due to the war crisis of Tigray, 2021 (*n* = 1,965)

Variables	Category	No Depression	With Depression
Age of HH head in Years	< 20	45 (36.3%)	79 (63.7%)
	21–35	213 (19.2%)	899 (80.8%)
	36–50	87 (15.6%)	469 (84.4%)
	> 50	22 (14.3%)	132 (85.7%)
Address of HH head	Southern	24 (16.4%)	122 (83.6%)
	South-eastern	25 (23.8%)	80 (76.2%)
	Mekelle	29 (31.2%)	64 (68.8%)
	Eastern	55 (17.4%)	261 (82.6%)
	Central	52 (28.1%)	133 (71.9%)
	North-western	61 (17.9%)	279 (82.1%)
	Western	110 (14.9%)	630 (85.1%)
Sex of HH head	Male	173 (19.7%)	703 (80.3%)
	Female	194 (17.9%)	889 (82.1%)
Marital Status of HH head	Single	113 (26.4%)	315 (73.6%)
	Married	228 (17%)	1114 (83%)
	Divorced/Widowed	27 (15.3%)	149 (84.7%)
Educational Status of HH head	No formal education	27 (15.8%)	144 (84.2%)
	Primary	96 (17.8%)	444 (82.2%)
	Secondary	136 (21.5%)	498 (78.5%)
	College +	49 (15.1%)	276 (84.9%)
The religion of HH head	Orthodox	364 (19%)	1551 (81%)
	Muslim	2 (5.1%)	37 (94.9%)
Occupation of HH head before displacement	Farmer	48 (16%)	252 (84%)
	Gov’t employee	57 (15.8%)	303 (84.2%)
	Private employee	19 (18.6%)	83 (81.4%)
	Merchant & Investor	108 (18.3%)	485 (81.7%)
	Daily labor	42 (17.1%)	203 (82.9%)
	Other^a^	92 (25.8%)	264 (74.2%)

^a^no job, student, housewife

### Prevalence of depression

**Fig. 1:**
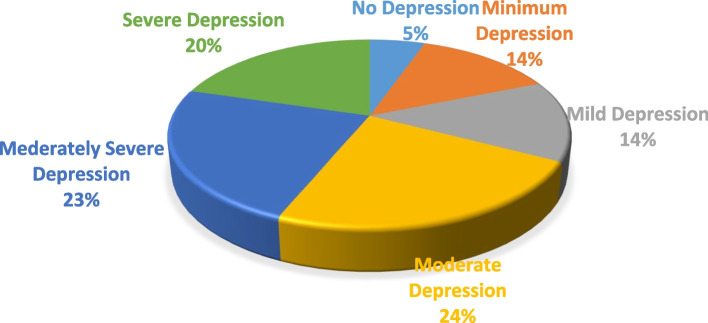
Classification of depression based on severity among cIDP due to war crisis in Tigray, 2021 (*n* = 1,965)

The prevalence of depression was 1,596 [ 81.2% (95% CI: 79.4–83%)]. Regarding the severity of depression; according to DSM-V classification around 67% of the participants were classified as having moderate to severe depression (Fig. 1).

The median estimated monetary value of looted and destroyed properties was 100,000 (IQR = 309,469) ETB. Around half 49.2% and 51.9% of the displaced population hosted in the community had family sizes of 4–6 and 5–8 respectively. Seventy-three percent of fixed assets and 65.6% of cereals were looted (by Eritrean, Ethiopian, and Amhara troops) during this war crisis. Among displaced Family sizes accounting 4–6 and > 6, 84.6% and 89.3% had depression consequently. From participants who have faced their fixed assets destroyed/burnt 87.7% of them developed depression. As participants' time stayed increased in months the prevalence of depression increased by 79.9%, 80.3%, and 81.5% respectively for < 4 months, 4 – 6 months, and > 6 months stay displaced in IDPs (Table 2).

**Table 2:** Level of depression and economic-related factors among cIDPs of Tigray due to the war crisis in Tigray, 2021 (*n* = 1,965)

Variable	Category	No Depression	With Depression
Displaced Family Size	1–3	215 (24.5%)	664 (75.5%)
	4–6	114 (15.4%)	624 (84.6%)
	> 6	18 (10.7%)	150 (89.3%)
Family size of host HH	1–4	145 (20.1%)	578 (79.9%)
	5–8	161 (18.3%)	719 (81.7%)
	9–12	17 (18.1%)	77 (81.9%)
How is the condition of your fixed asset	Normal	49 (27.7%)	128 (72.3%)
	Destroyed/Burnt	34 (12.3%)	243 (87.7%)
	Looted	185 (15.1%)	1040(84.9%)
Length of time stayed displaced in IDP	< 4 month	42 (20.1%)	167 (79.9%)
	4 – 6 months	47 (19.7%)	191 (80.3%)
	> 6 month	278 (18.5%)	1228(81.5%)
Shortage/Lack of necessities	No	33 (31.4%)	72 (68.6%)
	Yes	134 (15.7%)	719 (84.3%)
Looted property during the war	Yes	248 (16.1%)	1295(83.9%)
	No	119 (29.2%)	289 (70.8%)
Looted domestic animals	No	83 (19%)	354 (81%)
	Yes	32 (10.3%)	279 (89.7%)
Looted Machine/ house commodities	No	23 (19%)	98 (81%)
	Yes	86 (14.1%)	523 (85.9%)
Looted cereals	No	196 (30.5%)	447 (69.5%)
	Yes	156 (12.7%)	1072(87.3%)
The estimated monetary value of looted/damaged properties in ETB	< = 35,000	71 (16.9%)	348 (83.1%)
	35,001–100,000	79 (18.9%)	340 (81.1%)
	100,00–344469	56 (14.7%)	324 (85.3%)
	> = 344,470	47 (11.6%)	359 (88.4%)

Regarding the health status-related questions; Eighty-two percent of family members whose family had separated had depression, while around 86.1% of participants who had lost contact with family members had depression. Also, out of the participants who had lost family members, 87.2 had developed depression (Table 3).

**Table 3:** Level of depression and health status-related factors among cIDPs of Tigray due to the war crisis in Tigray, 2021 (*n* = 1,965)

Variables	Category	No-Depression	Have Depression
Separated family members	No	270 (19.1%)	1146 (80.9%)
	Yes	95 (18.1%)	429 (81.9%)
Lost contact with Family members	No	321 (20.1%)	1273 (79.9%)
	Yes	39 (13.9%)	241 (86.1%)
Family member death (*n* = 1882)	No	340 (20.2%)	1346 (79.8%)
	Yes	25 (12.8%)	171 (87.2%)
Family member faced disability	No	343 (20.5%)	1331 (79.5%)
	Yes	13 (8.6%)	139 (91.4%)
Family members with medical illnesses	No	305 (19.7%)	1247 (80.3%)
	Yes	43 (14%)	265 (86%)
Family members experience new health problems	No	298 (21.2%)	1105 (78.8%)
	Yes	54 (12.8%)	369 (87.2%)

### Factors associated with depression

Multiple factors were associated with depression in bivariate analysis. Variables with p < 0.25 were entered into the multivariable analysis (Table 4). In multivariable analysis, married respondents of IDP had 1.43 times (AOR = 1.43 95% CI 1.07–1.91) and divorced/widowed had 1.77 times (AOR = 1.77 95% CI 1.08–2.91) higher rate of depression than single respondents. Government employees were two times (AOR = 2.02 95% CI 1.27–3.2) more depressed compared to farmers. Similarly, daily laborers were 2.01 times (AOR = 2.01 95% CI 1.21–3.32) more depressed compared to farmers. Displaced people with family sizes 4–6 and > 6 family size was more depressed (AOR = 1.39 95% CI 1.05–1.83) and (AOR = 1.85 95% CI 1.07–3.2) respectively than those who have less than three family members. Participants with destroyed fixed assets (AOR = 1.75 95% CI 1.03–2.98) and looted cereals (AOR 2.12 95% CI 1.59–2.84) were more depressed than their counterparts. At last, cIDP who had a family member that faced disability due to the war crisis were 2.58 times (AOR = 2.58 95% CI 1.4–4.74) more depressed than those who didn’t face this problem (Table 4).

**Table 4:** Factors associated with depression among cIDPs of Tigray due to the war crisis in Tigray, 2021 (*n* = 1,965) (*n* = 1,965)

Variable name	Category	COR (CI-95%)	AOR (CI-95%)
Age of family head (Year)	< 20	1	1
	21–35	2.4(1.62–3.57) ***	1.2(0.72–1.85)
	36–50	3.07(1.99–4.23) ***	2.7(1.81–4.28)
	> 50	3.42 (1.91–6.11) ***	2.36(1.23–4.17)
Marital status	Single	1	1
	Married	1.75(1.35–2.67) ***	1.43(1.07–1.91) *
	Divorced/Widowed	1.98 (1.25–3.14) **	1.77(1.08–2.91) *
Educational Status	No Formal Education	1	1
	Primary	0.87(0.54–1.38)	1.8(0.54–1.76)
	Secondary	0.69 (0.44–1.08)	0.93(0.65–1.54)
	College and +	1.06 (0.63–1.76)	2.1(1.23–3.68)
Occupation	Farmer	1	1
	Gov’t Employee	1.01(0.67–1.54)	2.02(1.27–3.2) **
	NG-Employee	0.83(0.46–1.5)	1.7(0.9–3.23)
	Merchant	0.86(0.59–1.24)	1.35(0.9–2.04)
	Daily laborer	0.92(0.59–1.45)	2.01(1.21–3.32) **
	Other	0.55(0.37–0.81)**	1.18(0.76–1.83)
Displaced family size	1–3	1	1
	4–6	1.77(1.38–2.28) ***	1.39(1.05–1.83) *
	> 6	2.7(1.62–4.51) ***	1.85(1.07–3.2) *
Condition of Fixed Asset	Normal	1	1
	Destroyed	2.74(1.68–4.45) ***	1.75(1.03–2.98) *
	Looted	2.15(1.5–3.1) ***	1.37(0.9–2.09)
Shortage/Lack of necessities	No	1	1
	Yes	2.46(1.57–2.86) ***	1.86(1.25–3.32)
Property loss during the war	No	1	1
	Yes	2.15(1.67–2.77) ***	
Looted domestic animals	No	1	1
	Yes	2.04(1.32–3.16) **	1.57(0.97–2.56)
Looted Machineries/ House commodities	No	1	1
	Yes	1.43(0.86–2.37)	1.5(0.86–2.63)
Looted Cereals	No	1	1
	Yes	3.01(2.38–3.82) ***	2.12(1.59–2.84) ***
Lost contact with a family member	No	1	1
	Yes	1.56(1.09–2.23) *	1.09(0.83–2.42)
A family member died due to war	No	1	1
	Yes	1.73(1.12–2.67) *	1.38(0.87–2.2)
Family members faced disability	No	1	1
	Yes	2.76(1.54–4.92) **	2.58(1.4–4.74) **
A family member experienced an acute health problem	No	1	1
	Yes	1.84(1.35–2.52) ***	1.38(0.99–1.92)

*COR* Crude odds ratio, *AOR* Adjusted odds ratio, *CI* Confidence interval and 1: reference group

^*^p < 0.05, **p < 0.01, ***p < 0.001

## Discussion

Based on this study, the overall prevalence of depression was found 81.2%. The factors significantly associated with the outcome variable were married and divorced marital status, being government employee, having family size >  = 4, destruction of fixed assets, looting of cereals, and injury of a family member/s.

The finding of this study showed that the overall prevalence of depression among community-hosted internally displaced persons was 81.2%. This result is higher compared to the study conducted in different countries such as: 30.7% in Uganda [29], 50% in South Sudan [30], 59% in Somali [31], 38.3% in Southeast Ethiopia [32], 37.8% in Eritrean Refugees [33], and 26.4% in a pooled prevalence of 81 studies done using a systematic study [34]. In this study, this might be due nature/severity of the war and exposure to double burden/impact which are; facing different difficulties as a result of the war (death/injury to oneself or a family member, being displaced from their original area, losing their properties…) and being unable to even get humanitarian aid from government and international community that would help them survive for some time due to the complete siege put on Tigray [18, 19]. The high prevalence of depression among the sampled population sounds for structured interferences to deal with mental health problems. And also it implies the need for more study (preferably qualitative) on the mental health issues in this population.

Regarding factors associated with depression, divorced/widowed /married displaced household heads were more depressed than single ones. This finding is similar to the study done in Uganda [13] and Southern Sudan [30]. This might be due to having additional responsibility and thinking about family members and other household administrations. In addition to this, they are more vulnerable to facing financial problems since they lost their properties, have no cash, no electricity, no transport, no communication, and other public infrastructures that help the daily survival life of them and their families [25, 31, 34, 35].

The other significant factor associated with depression was the occupational status of the cIDPs. Unemployment is also more associated with depression according to some studies done on Somali refugees [36], and Eritrean refugees [32]. In this study government employees and daily labourers were more depressed than farmers. This is a unique finding which might be due to the absence of salary and the deliberate closing of banks making it hard for government employees to even access what they had in their accounts, and their source of money/expenditure; not having it, especially when they have a family to take care of, makes it hard for them and puts them in a depressive state. Similarly, due to the current absence of construction or other jobs which require labor, the absence of transactions at the previous level. In general, may the war made it hard for daily laborers to feed themselves and their families and puts them in depression [23].

Family size was also found to be a significant factor for depression in this study, those with 4 or more family sizes were more depressed than those who had less family size, this is still believed to be associated with financial issues i.e. the bigger family size they have the more they are going to struggle with financial issues; which is hard due to the complete siege that there was no banks, no communication lines, no sufficient resources for daily utility use throughout Tigray. In addition to this, there was not farming during the whole war period, no transport in and out of Tigray and the uncertainty about for how long it is going to last worries the people.

In addition to this, cIDPs who were looted of their cereals, destructed of their fixed assets, and lost of their property were found to be associated with depression. This finding is consistent with a study done in Croatia [6]. This is obvious that, where ever in the world a person lives, as a human being, it is hard when one loses everything they have worked for and their home in a blink of an eye due to the war they didn't expect. Displacement deteriorates health outcomes, increase loss of employment, and inability to access public services. This may result in hopelessness and loss of one's direction.

Injury of family members due to the war in Tigray was also another factor associated with depression. This is similar to studies done in Northern Uganda [13], Islamabad-Pakistan [33], and Croatia [6] where war stressors like being seriously injured or having a close family member who got injured or experiencing the death of close persons were found to be associated with depression. Even though resilience and how a person deals with a stressful situation; hence whether a person becomes depressed as a result of stressors differs from one person to another, it's no surprise that one might develop symptoms of depression as a result of lasting injuries of trauma more compared to those who were in a war situation but didn't experience such lasting effects of the war.

### Strengths and limitations of the study

This study was done in the all *woredas*/districts of Tigray (with a large sample size) at the community level and this may have external validity and generalization for internal and other IDPs globally. However, as a limitation of this study; since the study was cross-sectional it doesn't create a temporal causal relationship between the outcome variable with the significant factors. Also, this study focused on interviewing only the HH heads of the cIDPs so the other members were not interviewed which might have underestimated the finding of the study.

## Conclusions

The prevalence of depression among community internally displaced people during the war on Tigray is very high compared to other studies conducted elsewhere. Almost 8 out of 10 IDPs developed depression and this is a very series health issue and needs immediate psycho-social health intervention by local and international organizations with the collaboration of governmental and non-governmental institutions based on the study's findings.

## Data Availability

All the data supporting the findings is contained within the manuscript, when there is in need the data set used for the present study's conclusion can be accessible from the corresponding author upon reasonable request.

## Abbreviations

cIDPs: Community hosted Internally Displaced Persons
HH: Households
PHQ: Patient Health Questionnaire
SPSS: Statistical Package for Social Sciences
CI: Confidence interval
DSM: Diagnostic Statistical Manual for mental health disorder
IQR: Inter Quartile Range
MDD: Major Depressive Disorder
TBH: Tigray Bureau of Health
VIF: Variance Inflation Factor
